# Daily occupational exposure in swine farm alters human skin microbiota and antibiotic resistome

**DOI:** 10.1002/imt2.158

**Published:** 2024-01-01

**Authors:** Dong‐Rui Chen, Ke Cheng, Lei Wan, Chao‐Yue Cui, Gong Li, Dong‐Hao Zhao, Yang Yu, Xiao‐Ping Liao, Ya‐Hong Liu, Alaric W. D'Souza, Xin‐Lei Lian, Jian Sun

**Affiliations:** ^1^ State Key Laboratory for Animal Disease Control and Prevention South China Agricultural University Guangzhou China; ^2^ Guangdong Laboratory for Lingnan Modern Agriculture, National Risk Assessment Laboratory for Antimicrobial Resistance of Animal Original Bacteria, College of Veterinary Medicine South China Agricultural University Guangzhou China; ^3^ Guangdong Provincial Key Laboratory of Veterinary Pharmaceutics, Development and Safety Evaluation South China Agricultural University Guangzhou China; ^4^ Jiangsu Co‐Innovation Center for the Prevention and Control of Important Animal Infectious Disease and Zoonoses Yangzhou University Yangzhou China; ^5^ Veterinary Center Guangxi State Farms Yongxin Animal Husbandry Group Co., Ltd. Nanning China; ^6^ Laboratory Animal Centre Wenzhou Medical University Wenzhou China; ^7^ Department of Pediatrics Boston Children's Hospital Boston Massachusetts USA; ^8^ Harvard Medical School Boston Massachusetts USA

**Keywords:** antibiotic resistome, metagenomic sequencing, occupational exposure, skin microbiota, swine farm

## Abstract

Antimicrobial resistance (AMR) is a major threat to global public health, and antibiotic resistance genes (ARGs) are widely distributed across humans, animals, and environment. Farming environments are emerging as a key research area for ARGs and antibiotic resistant bacteria (ARB). While the skin is an important reservoir of ARGs and ARB, transmission mechanisms between farming environments and human skin remain unclear. Previous studies confirmed that swine farm environmental exposures alter skin microbiome, but the timeline of these changes is ill defined. To improve understanding of these changes and to determine the specific time, we designed a cohort study of swine farm workers and students through collected skin and environmental samples to explore the impact of daily occupational exposure in swine farm on human skin microbiome. Results indicated that exposure to livestock‐associated environments where microorganisms are richer than school environment can reshape the human skin microbiome and antibiotic resistome. Exposure of 5 h was sufficient to modify the microbiome and ARG structure in workers' skin by enriching microorganisms and ARGs. These changes were preserved once formed. Further analysis indicated that ARGs carried by host microorganisms may transfer between the environment with workers' skin and have the potential to expand to the general population using farm workers as an ARG vector. These results raised concerns about potential transmission of ARGs to the broader community. Therefore, it is necessary to take corresponding intervention measures in the production process to reduce the possibility of ARGs and ARB transmission.

## Highlights

- Exposure to livestock‐associated environment, more microbially enriched than school environment, can remodel the human skin microbiome and antibiotic resistome.
- The 5 h of exposure is sufficient to alter the microbiota and antibiotic resistance genes (ARGs) structure of skin and these changes were retained once formed.
- ARGs carried by host microbes were transferred between the environment and workers' skin.

## Graphical abstract

We show that occupational exposure in a livestock environment dynamically reshaped the skin microbiome and resistome. This remodeling effect can take place within hours and the acquired pathogens and antibiotic resistance genes (ARGs) have the potential to expand to the general population using farm workers as an ARG vector. Farm workers deserve special consideration under the One Health framework to curb spreading antibiotic resistance.

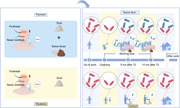

## INTRODUCTION

Emergence and spread of antimicrobial resistance (AMR) pose serious global public health danger. Given the interdependence of AMR for humans, animals, and the environment, a “One Health” approach is imperative. Due to high antibiotic utilization, livestock breeding environments are highlighted as hotspots for antibiotic resistance genes (ARGs) and antibiotic resistant bacteria (ARB). The scientific basis for this study to focus on environmental exposure in swine farm is mainly based on the following reasons. The first reason is that animal husbandry represents an intermediate interface between the natural environment and humans [1], and it is of great significance to elucidate how animal husbandry bridges the gap between the environment and the human microbiome. The second reason is that animal husbandry has abundant occupational exposure factors. Workers' skin microbiota may be easily disturbed by environmental microbes through direct contact with animals, bedding materials, feeding facilities, and especially airborne microbial communities [2, 3, 4]. It has been reported that the air of livestock farms, especially swine farms, is full of a large amount of dust, bacteria and fungi [5, 6], the concentration of bacteria in the air can reach 2 × 10^7^ [7], which is twice the level usually measured in indoor air. In addition, animal husbandry generally requires higher health standards to minimize economic losses caused by infectious diseases. The extensive use of disinfectants, antimicrobials, and preservatives, which may alter human microbiome patterns [8, 9, 10]. The third reason is that the abuse of antimicrobials in animal husbandry has brought great pressure on human and natural microbial systems, a circulation chain of ARG transmission exists between livestock and humans [11] which impacts the surrounding environment [12] that cause the environment of animal husbandry farms be usually rich in a large amount of ARGs and ARB [13, 14, 15]. Thus, studying the distribution and transfer patterns of ARGs and ARB in humans, livestock, and farming environments is critical to mitigate spreading AMR.

Skin is the largest organ in the human body, and it provides ecological niches for microorganisms [16], including bacteria, archaea, fungi, and viruses, collectively known as the skin microbiome [17]. Microorganisms with high abundance and long‐term colonization on the skin are in a long‐term equilibrium state, and microflora with medium or low abundance are most prone to be changed [18]. The composition and diversity of skin microbial communities are affected by factors, including intrinsic factors like age [19, 20], gender [21], genetic variation [22], and external interference such as lifestyle, daily behavior, and environmental exposures. Behaviors such as cleaning and disinfection can change skin microbial community composition but mainly affect transient skin microorganisms [23, 24, 25]. Studies also show that microbiota can be transferred from the environment to human skin. For example, people living near rural and forest environments have higher microbial diversity on their skin compared to urban environments [26]. Exposure to urban green spaces may improve human health by altering human microbiota composition and increasing microbial diversity in the skin and nasal cavity [27]. Ahn et al., [28] discussed these macroenvironmental factors in a review and highlighted that work‐related occupational exposures were common [29]. Occupational exposure alters workers' skin microbiome and may threaten their health. A study on the skin microbiome of staff in Romania museum showed over half of study subjects have showed a low rate of skin fungal colonization and some ARB has been detected in the skin microbiome [30]. Studies on farmworkers' skin microbiota in the United States demonstrated that high‐density farm animal handling could lead to changes in the skin microflora of human forearms, increasing the risk of skin infection of farmworkers with unusual pathogens and epidermal diseases [31]. Sanitation procedures can be an effective method of removing transient microorganisms from the skin procedures [32]. Most of swine farms including our study site require workers to take sanitation measures before entry due to the prevalence of veterinary pathogens like African Swine Fever Virus (ASFV), *Mycoplasma hyopneumoniae*, and *Streptococcus suis* in China [33, 34, 35, 36].

A number of recent studies have focused on the effects of environmental exposure on the oral or gut microbiome and antibiotic resistome of workers in farms and the transmission of ARGs between the gut and the environment [37, 38]. The human skin microbiota has higher variability compared to more stable gut or oral microbial communities [39, 40], it's also an important ARG reservoir [41]. The characteristics and extent of ARGs and ARB transmission between the farming environment and human skin are largely unknown. Therefore, the impact of environmental exposure in swine farms on the human skin microbiome and antibiotic resistome is of interest. One common approach for skin microbiome investigation studies is amplicon sequencing [42, 43, 44]. This method simplifies computational analysis, but it also has some disadvantages [45]. For example, variable region selection or primer selection can have a profound impact on sequencing results. The resolution of taxonomic profiles is low, and most terminates at the genus level [46, 47]. Detailed skin microbiological research requires more accurate and complete microbial genome information for functional analysis and differentiation of different strains. These goals are better achieved by metagenomic sequencing.

We compared metagenomic sequencing samples from students and swine farm workers to understand the impact of daily swine farm environmental exposures on the human skin microbiome and antibiotic resistome. We specifically focused on exposure timeline to clarify the time course of microbiota and resistome changes following exposure. Our investigations revealed exposure of 5 h has been sufficient to modify the microbiome and ARG structure in workers' skin. We also explored the mechanism of ARGs transmission between the swine farm environment and workers' skin. These findings provided insights into the potential risk of ARGs transmission to the broader community and highlight opportunities for public and occupational health interventions.

## RESULTS

### Metagenome sequencing and quality control

**Figure 1:**
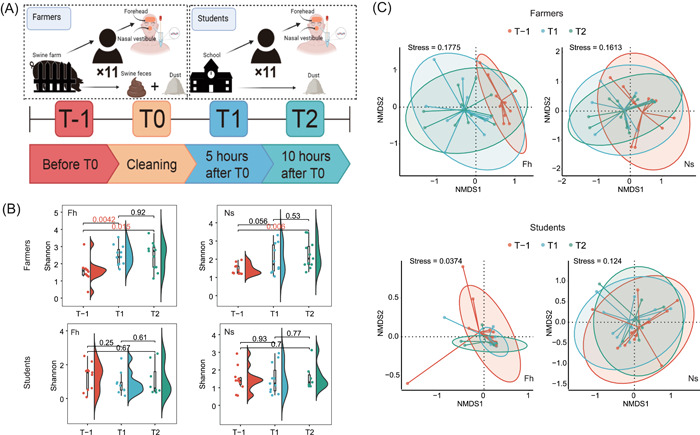
Changes in skin microbiota diversity following environmental exposure. (A) Experimental design schematic: Farmers, eleven swine farm workers; Students, eleven university students; T‐1 pre‐exposure, T0 skin cleaning, T1 5 h postexposure, and T2 10 h postexposure. (B) Changes in microbial diversity (Shannon diversity index) of samples from groups Fh and Ns of two cohorts (Farmers and Students) at T‐1, T1, and T2. *p* values marked in red mean are less than 0.05. (C) Nonmetric multidimensional scaling (NMDS) analysis of the microbial community structures in group Fh (forehead) and group Ns (nose) of two cohorts based on Bray–Curtis dissimilarity at T‐1 (red), T1 (blue), and T2 (green).

We collected and sequenced 176 skin samples and 128 environmental samples from workers and students to profile the dynamics of the skin microbiota and antibiotic resistome after daily occupational exposure (Figure 1A). Timepoint 0 (T0) samples collected following skin cleaning had low DNA yields for all samples and were not detectable by Qubit (in 50 μL of eluted sterile water <0.001 ng/μL), and thus they could not be sequenced for library construction. Additional 27 samples were not sequenced successfully. A total of 121 human skin and 111 environmental samples were successfully sequenced and available for subsequent analysis. The quality metrics of these sequenced samples are shown in Table S3.

### Skin microbiota diversity increased with environmental exposure

To understand the effect of daily occupational exposure on the diversity of skin microbiota in healthy individuals, we analyzed the microbial communities of forehead skin (Fh) and nasal vestibule skin (Ns) swab samples from workers and students. The alpha diversity of workers Fh and Ns microbial communities were significantly increased (*p* = 0.0042) and showed a strong upward trend (*p* = 0.056) 5 h after exposure, roughly doubling the Shannon index. In contrast, no significant change occurred in workers skin samples from 5 to 10 h of exposure (Fh: *p* = 0.92, Ns: *p* = 0.53). Students' skin microbiota diversity remained stable throughout a day (Figure 1B and Figure S1).

The skin microbial community structure of workers and students was characterized using nonmetric multidimensional scaling (NMDS) and Permutational multivariate analysis of variance (PERMANOVA) analysis (Figure 1C). In workers, the microbial community structure of Fh changed significantly from Timepoint‐1 (T‐1) to Timepoint 1 (T1) (R^2^ = 11.8%, *p* = 0.01) and insignificantly from T1 to Timepoint 2 (T2) (R^2^ = 6.9%, *p* = 0.169). The microbial community structure of group Ns changed but not significantly from T‐1 to T1 (R^2^ = 6.2%, *p* = 0.260) and barely changed from T1 to T2 (R^2^ = 1.5%, *p* = 0.943). In students, the microbial community structure of groups Fh and Ns from T‐1 to T1 (Fh: R^2^ = 5.5%, *p* = 0.321; Ns: R^2^ = 4.1%, *p* = 0.536) and from T1 to T2 both had no significant change (Fh: R^2^ = 3.6%, *p* = 0.610; Ns: R^2^ = 1.7%, *p* = 0.928). These results indicate that skin microbial communities exposed to swine farm environments can rapidly achieve steady state. In contrast, the skin microbial community structure of the students did not change significantly within a day.

### Skin microbiota composition changes correlate with occupational exposure in swine farm

To understand the taxonomic changes underlying the alpha diversity and broad compositional changes revealed in Figure 1, we investigated the workers' skin microbiota at the phylum, genus, and species levels. Our taxonomic analysis identified 25 phyla, 391 genera, and 1189 species of archaea, bacteria, and fungi (Table S4).

**Figure 2:**
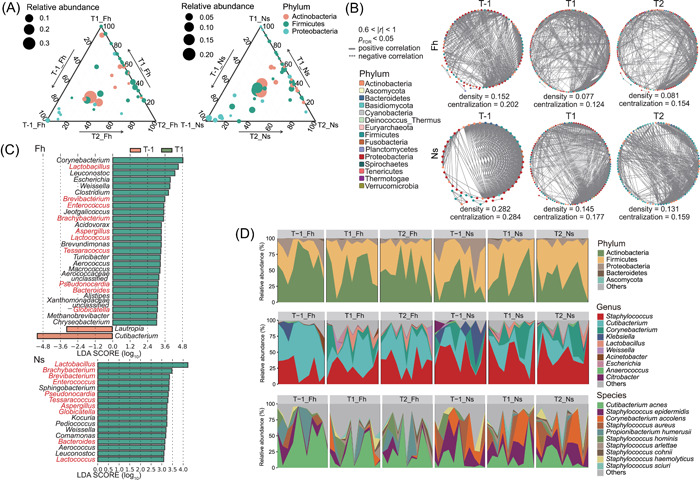
The variation of workers' skin microbiota composition across time points. (A) Ternary plot showing taxa abundance changes in samples from Fh and Ns during T‐1, T1, and T2. Dot size indicates the average abundance of the genus in the sample, and dot color indicates their corresponding phylum. (B) Microbial co‐occurrence networks on genus level demonstrated reduced inter‐genera correlation after exposure. (C) LEfSe analysis for characteristic microbial genera at T‐1 and T1. Only results with Linear discriminant analysis (LDA) score (log _10_) > 3 are shown. (D) Stacked area plots showing relative abundance of microbes at the phylum, genus, and species resolutions. The label on the X‐axis was omitted is sample name.

Before occupational exposure, the forehead skin microbiome of workers was dominated by Actinobacteriota, while the nasal vestibular skin microbiome was dominated by Firmicutes, accounting for 61.09% and 49.35% respectively. As shown in Figure 2D and Table S5, the dominant phyla (top three in terms of sum of abundance) remained similar before and after exposure with Proteobacteria from nasal vestibular skin having the only significant relative abundance change (T1/T‐1: *p* < 0.05).

The ternary diagram (Figure 2A) shows the comparative abundance of skin microbiota at the genus level across timepoints. Overall, the dominant genera were mainly from three phyla, Actinobacteria, Firmicutes, and Proteobacteria, and the dominant genera changed slightly after exposure, with the dominant genera at T‐1 being Actinobacteria, while at T1 and T2 there was an enrichment of genera attributed to Actinobacteria and Proteobacteria. For each sample type, the spearman correlation coefficient was used to describe the neighborhood relationships between genera. By comparing the correlated co‐occurrence networks of the skin microbiota in groups Fh and Ns across timepoints (Figure 2B), both groups showed a weaker intergenera correlation and a sharp decrease in network density (used to describe the fraction of potential connections between microorganisms) and network centralization (used to measure the dispersion of the centrality scores of all nodes in the network in relation to the maximum centrality score obtained in the network) at T1 compared to T‐1. We used Linear discriminant analysis Effect Size (LEfSe) analysis to identify changes in the characteristic microbial genera before and after occupational exposure. The relative abundance of 10 genera including *Lactobacillus*, *Brevibacterium* and *Enterococcus* both in groups Fh and Ns at T1 were significantly higher than at T‐1 (Figure 2C).

Species with significant changes in forehead skin were *Staphylococcus arlettae*, *Staphylococcus cohnii*, *Staphylococcus sciuri* and *Corynebacterium xerosis*, and species with significant changes in nasal vestibular skin were *Staphylococcus arlettae*, *Staphylococcus sciuri*, and *Corynebacterium xerosis*. Significant changes in relative abundance were found for low abundance species (less than 0.5%) at T‐1, while high abundance species remained stable. Results of taxonomy annotation at the species level are provided in Table S7 and are shown in the stacking area plot (Figure 2D).

### Microbial transmission from the swine farm environment to workers' skin

**Figure 3:**
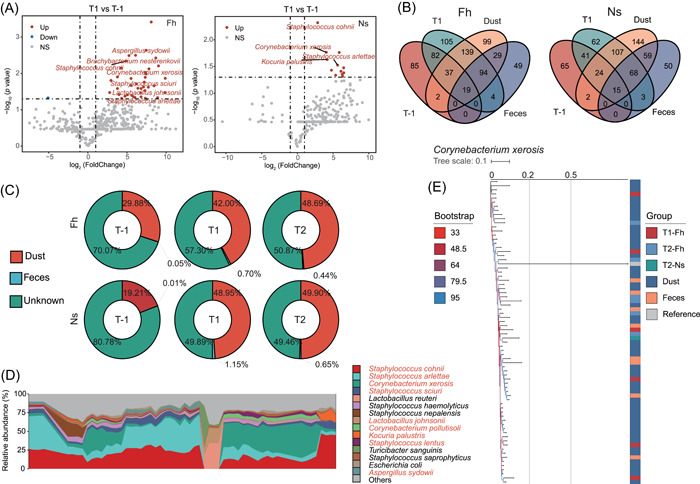
Transmission of microbes from the swine farm environment to workers' skin. (A) Volcano plots showing alteration of microbiota composition on species level of forehead and nasal vestibular skin after 5 h of occupational exposure with the *x* axis denoting log_2_(Fold Change) and the y‐axis denoting −log_10_ *(p* value). Significantly increased species at T1 are red and significantly decreased species are blue. Dashed vertical and horizontal lines reflect the filtering criteria (absolute fold‐change (FC)  ≥  1.0 and *p* < 0.05). (B) Venn diagram quantifying shared and unique microbial species between dust samples and samples from three time points of forehead and nasal vestibular skin. (C) Source prediction of skin microbiota during T‐1, T1, and T2. (D) Stacked area plot showing the top 15 relative abundant species in dust. Nine of the top fifteen species in relative abundance were the significantly changed species we previously identified. The label on the *x* axis was omitted is sample name. (E) Phylogenetic tree of *Corynebacterium xerosis* at strain‐level using StrainPhlAn3. The reference genome of *Corynebacterium xerosis* is from *Corynebacterium xerosis* ASM364124v1. Bootstrap support values are indicated by the color of legend.

Comparing before and after exposure timepoints, we identified 41 significantly changed species from forehead samples and 12 significantly changed species from nasal skin samples including *Corynebacterium xerosis*, *Corynebacterium amycolatum*, and *Staphylococcus lentus* among others (Figure 3A and Table S8). Several of these species are opportunistic pathogens, and all except *Streptococcus sanguinis* were enriched after 5 h of exposure. These results are consistent with our finding that environmental exposure resulted in changed richness and evenness of dermal microbial communities in workers. 11 of the species significantly different from pre and post exposure were shared by the groups Fh and Ns. The change in relative abundance of these species was greater in forehead skin microbiome following daily occupational exposure than the nasal vestibular skin (Figure S2A). Though these changed species differed in their relative abundance percentages at the individual level, they were all less than 0.5% at T‐1, and the trends produced by environmental exposure were generally consistent (Figure S2B).

Additionally, Figure 3B shows how the number of microbial species shared by the environment and the workers' skin changed after short‐term exposure. From T‐1 to T1, the species shared by workers' skin with the environmental microbiome increased significantly, and the species shared by workers with the dust microbiome was consistently much greater than the species shared with the fecal microbiome. The number of species shared with dust in the group Fh increased from 58 (7.7%) to 289 (38.6%), while the number of species shared with dust in the group Ns increased from 41 (6.9%) to 214 (35.5%). Sourcetracker analysis assessing association between skin microorganisms and environmental microorganisms (Figure 3C) showed that 29.88% of forehead skin microbial composition was associated with dust microorganisms at T‐1. This proportion increased to 42.00% at T1% and 48.69% at T2. For nasal vestibular skin samples, 19.21% of microbial composition was associated with dust microorganisms at T‐1, which increased to 48.95% at T1% and 49.90% at T2. Skin microbial composition was minimally affected by fecal microorganisms across time points, and the proportion was less than 1% in each group apart from nasal vestibular skin at T1. Though this analysis identified many species level associations, many bacterial source associations remain unidentified and it is unclear if they originate from other environmental factors in farm. We examined the microbial composition of the dust samples and found 9 of the top 15 species in relative abundance were the significantly changed species we previously identified (Figure 3D). These results suggest that dust microbes play a more important role in the environmental impact on skin microbiota than swine feces.

To gain more insight into the potential commonalities between the changed skin microbiota and the environmental microbiota, we conducted an analysis of the population structure at the level of individual strains. Eight significantly changed species co‐occurred in skin after exposure and in dust samples with close phylogenetic relationships (with a normalized phylogenetic distance of no more than 0.1) (Figures S3 and S4). One of these, *Corynebacterium xerosis* (Figure 3E) is most prevalent, and it is an opportunistic pathogen present in human and animal skin mucosa capable of causing endocarditis, skin infections and other diseases.

### Antibiotic resistome structure influenced by changing environment

To assess if microbiota changes were correlated with ARG changes, we analyzed the workers' skin antibiotic resistome and detected 20 ARG types representing 618 ARG subtypes (Table S9).

The skin antibiotic resistome had significantly higher shannon diversity at T1 compared to T‐1 (Fh: *p* = 0.016, Ns: *p* = 0.023), but it remained stable from T1 to T2 (Fh: *p* = 0.99, Ns: *p* = 0.96) (Figure S5). NMDS and PERMANOVA analysis showed that there was a significant change in the structure of the skin antibiotic resistome from T‐1 to T1 (Fh: R^2^ = 24.2%, *p* = 0.01; Ns: R^2^ = 8.9%, *p* = 0.033) and no significant change from T1 to T2 (Fh: R^2^ = 3.9%, *p* = 0.624; Ns: R^2^ = 2.0%, *p* = 0.945). The skin antibiotic resistome after exposure overlapped with the dust antibiotic resistome and partially overlapped with the swine feces antibiotic resistome, suggesting that the distributions of skin and dust ARG profiles were relatively similar and differs somewhat from skin with swine feces. Diversity of the antibiotic resistome in students' skin did not change significantly within a day. Overall, the skin ARG diversity profile correlated with microbiome diversity.

**Figure 4:**
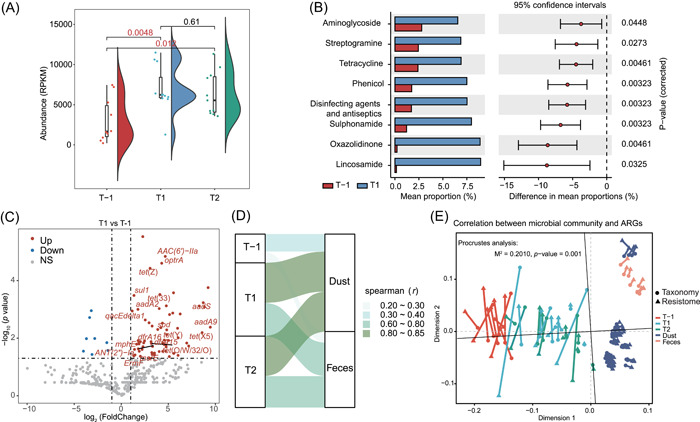
Antibiotic resistome structure was influenced by occupational exposure. (A) Sum of forehead skin antibiotic resistance gene (ARG) abundance across T‐1, T1 and T2. Boxes show the distribution of workers' samples (*n* = 10/11 biologically independent samples per time point) (boxes show medians/quartiles; error bars extend to the most extreme values within 1.5 interquartile ranges). *p* values in red are less than 0.05. (B) Comparison of relative abundance of ARG types in forehead skin. Statistics were conducted by the Student's T‐test and Benjamini–Hochberg FDR correction. (C) Volcano plots showing the alteration of distribution of ARG subtypes in forehead skin after 5 h of occupational exposure. The log_2_FoldChange were used to illustrate the variation of ARGs at T1 compared with T‐1. The red/blue dots represented the ARGs significantly increased/decreased in T1 compared to T‐1. Dots marked with text represent the common ARGs from groups Fh and Ns enriched at T1. (D) Sankey plot showing correlation between the ARG composition of forehead skin samples across time points and the ARG composition of environmental samples. The height of the rectangles and the depth of color all indicate the Spearman correlation between human skin microbime and swine farm environment microbiome. (E) Procrustes analysis connecting the microbiomes and resistomes of microbiota in forehead skin and environmental samples.

Compared to pre‐exposure, the sum of the relative ARG abundances (mean reads per kilobase of reference sequence per million sample reads, RPKM) increased sharply (*p* = 0.0048) with a mean increase of 127.14% in the forehead skin (Figure 4A) and slightly (*p* = 0.15) with a mean increase of 4.01% in the nasal vestibular skin (Figure S6A). The ARG type with the most significant increase in abundance in forehead skin postexposure compared to pre‐exposure was lincosamide, followed by oxazolidinone and sulphonamide, with no significant downregulation of ARG types (Figure 4B). There were no significant changes in ARG types in nasal vestibular skin. To identify ARG subtypes where abundance changed significantly, we performed pre and post exposure differential analysis. The ARG subtypes significantly enriched after 5 h of exposure mainly belong to tetracycline resistance gene, including *tet*(X), *tet*(X3‐X6), *tet*(W) and 25 other subtypes, followed by aminoglycoside resistance gene including *AAC(6’)_Ie_APH(2”_Ia*, *ANT(9)‐Ia*, *aadA2* and 21 other subtypes and Macrolide‐Lincosamide‐StreptograminB (MLSB) resistance gene, with 13 subtypes including *ErmB*, *ErmF*, *ErmT* etc (Figures 4C, S6B and Table S10).

### Transfer of ARGs between the swine farm environment and human skin

**Figure 5:**
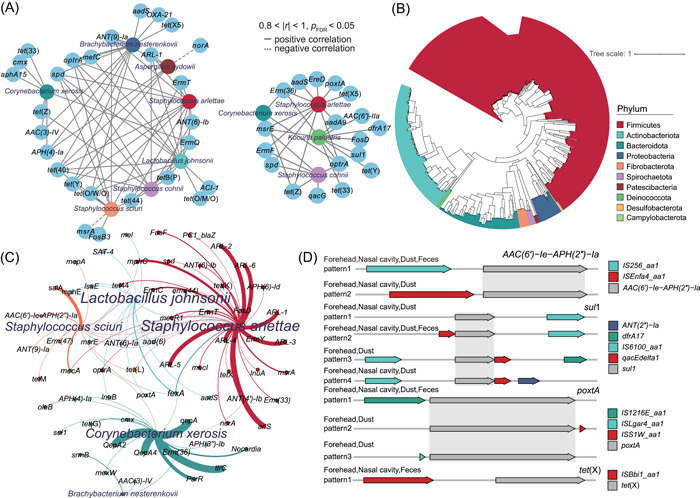
ARGs were transferred via microbes among swine farm environment and human skin. (A) Network analysis of co‐occurrence between changed ARGs and microbes transmitted from environment (*p* < 0.05, absolute correlation coefficient >0.80). (B) Phylogenetic assignment of metagenome‐assembled genomes (MAGs). Organisms are colored based on phyla. (C) Network analysis of co‐occurrence patterns between major MAGs (changed microbes) and ARG subtypes. Nodes are colored according to species. Node sizes are proportional (average weighted) to the number of connections. The width of the curves represented the abundance of ARGs carried by their hosts. (D) Genomic structure patterns of mobile ARG patterns present in both environment and human samples. Four ARGs *AAC(6’)_Ie_APH(2”)_Ia*, *sul1*, *poxtA*, and *tet*(X) are illustrated and aligned for each pattern, ARGs are shaded in grey.

Environmental and skin ARG profiles measured by spearman correlation coefficients increased significantly after 5 h of exposure and remained stable from 5 to 10 h (Figures 4D and S6C). Specifically, the correlation coefficients for forehead skin with dust at T‐1 was *r* = 0.35, with feces was 0.23; at T1, the correlation coefficients for forehead skin with dust was *r* = 0.80, with feces was 0.67; at T2, the correlation coefficients for forehead skin with dust was *r* = 0.80, with feces was 0.63. The results for group Ns are essentially the same as group Fh and the implying that ARGs in dust may play an important role in the variation of skin antibiotic resistome, mirroring the microbial results. Procrustes analysis based on Bray‐Curtis distances revealed a good fit and significant correlation between the entire ARG profile and microbial community composition (Fh: M^2^ = 0.2010, *p* = 0.001; Ns: M^2^ = 0.1388, *p* = 0.001) (Figures 4E and S6D). These results suggest that the workers' skin antibiotic resistome is consistent with change trends in their microbiome, implying that changed ARGs may be from changed microorganism. Further analysis supported this hypothesis, we conducted Spearman's correlation analysis on ARGs with microorganisms that changed significantly after exposure, and selected highly significant and strongly correlated (0.8 < |*r* | < 1, *p*_FDR_ < 0.05) relationships to demonstrate the co‐occurrence network. As shown in Figure 5A, there are 8 significantly enriched microorganisms shared by skin and environment that were also potential hosts for significantly enriched ARGs were found. To further verify the presence of shared hosts carrying ARGs in workers' skin and the environment, we performed metagenomic assembly and binning of environmental samples to create a total of 2338 metagenome‐assembled genomes (MAGs). After screening for their completeness and contamination, a total of 581 MAGs with ≥50% completeness and ≤10% contamination remained. These MAGs were annotated to species by GTDB‐TK and subjected to phylogenetic tree analysis, and they belong to 10 bacterial phyla, the most abundant of which was Firmicutes (58.7%), followed by Actinobacteriota (20.7%), Bacteroidetes (13.1%), and Proteobacteria (3.6%) (Figure 5B). 455 of the 581 MAGs were successfully annotated to the species level including *Corynebacterium xerosis* (*n* = 29), *Staphylococcus arlettae* (*n* = 21), *Staphylococcus sciuri* (*n* = 14), *Lactobacillus johnsonii* (*n* = 8) and *Brachybacterium nesterenkovii* (*n* = 3), all of which were differential species that underwent significant enrichment after exposure and co‐occurred in skin and environmental samples. ARG annotation of these MAGs screened a total of 76 hosts carrying multiple ARGs, 15 of which were enriched after exposure, such as *sul1*, *optrA*, *poxtA* and *tet*(X) (Figure 5C and Table S11). Some of the results were consistent with those obtained from the network analysis approach above: *Staphylococcus arlettae* was a potential host for *tet*(44), *optrA*, *spd*, *ARL‐1*, *ErmT*, *ANT(9)‐Ia*, and *ANT(6)‐Ib*. *Corynebacterium xerosis* was a potential host for *cmx* and *APH(4)‐Ia*. *Staphylococcus sciuri* was a potential host for *ANT(9)‐Ia*. This demonstrates the plausibility and accuracy of the localization of the host carrying ARGs in this study.

As in our previous study [38], contigs with both ARGs and MGEs were categorized as mobile ARGs. A length limit of 5 kb was then applied between ARGs and MGEs to restrict the analysis to ARGs located near MGEs, and 25 ARGs associated with different mobile ARG patterns and co‐exist in both skin and environmental samples were identified, such as *AAC(6’)‐Ie‐APH(2”)‐Ia*, *poxtA*, *sul1* and *tet*(X) which were also the ARGs enriched after occupational exposure (Figure 5D and Table S2). *AAC(6’)‐Ie‐APH(2”)‐Ia* gene encodes the aac(6’)‐Ie‐aph(2”)‐Ia enzyme, which confers resistance to gentamicin and almost all clinically aminoglycosides except streptomycin. The mobile *AAC(6’)‐Ie‐APH(2”)‐Ia* has two distinct mobile ARG patterns: (i) insertion sequence IS*256*; (ii) insertion sequence IS*431R*. The *poxtA* gene is a transferable oxazolidinone resistance gene that has been reported to be associated with florfenicol residues used to treat respiratory and intestinal bacterial infections in livestock and poultry. Mobile *poxtA* gene has three different mobile ARG patterns: (i) insertion sequence IS*1216E*; (ii) insertion sequence IS*S1W* and (iii) IS*Lgar4*. *Sul1* and *tet(X)* were commonly used veterinary ARGs in swine farms, and the mobile *sul1* gene was associated with four patterns and was the most diverse mobile ARG.

## DISCUSSION

The main phyla of healthy human skin microbial communities are Actinobacteria, Firmicutes, Proteobacteria, and Bacteroidetes [48, 49, 50]. The skin microbial communities in this study were also dominated by these four phyla before/after exposure, validating the representativeness and appropriateness of the methods used and the specimens collected. Trends in the forehead and nasal vestibular skin microbiota after exposure corresponded, but nasal vestibular skin changes were more limited, indicating higher stability than the forehead skin microbiota. This discrepancy is likely due to the lower exposure to the external environment [51]. The structure and diversity of the skin microbiota of farmworkers changed somewhat and reached a steady state after 5 h of occupational exposure, while the skin microbiota of students remained stable throughout the day. Daily occupational exposures in swine farms dynamically alters human skin microbiomes. Compared with other environments, the livestock industry is enriched with diverse microorganisms and even 1 day of exposure elicits alteration in skin microbiome. The results of the LEfSe analysis showed that the three characteristic genera with the highest LDA scores after 5 h of exposure were *Lactobacillus*, *Brevibacterium*, and *Enterococcus*. *Enterococcus* and *Lactobacillus* are ubiquitous in the intestinal flora of human and animals [52], so they are common in environments contaminated with human and animal feces. *Brevibacterium* were frequently found in dust in a variety of environments [53, 54, 55], so enrichment of these genera in the skin of workers through daily work or dust transmission is not surprising. Opportunistic pathogens such as *Corynebacterium xerosis* were also significantly enriched after exposure. *Corynebacterium xerosis* was found in human and animal skin mucosa that can cause endocarditis, skin infections, and other diseases [56, 57, 58, 59]. *Corynebacterium amycolatum* is a potential multidrug‐resistant opportunistic pathogen and causative agent of serious infections in human and animals [60], especially when involved in the nasal environment of immunocompromised patients. *Staphylococcus lentus* is a nasal pathogen that can cause sinusitis [61]. However, we also detected bacteria, such as *Staphylococcus cohnii*, which is a potentially beneficial skin commensal that can alleviate skin inflammation [62]. Our results indicate that dust rather than animal waste is the primary driver of skin microbiota changes following swine farm exposure, suggesting that such particles bridge microbiota exchange from animals to human. A similar result was found in a study where indoor dust microbial communities could inter‐transfer with human skin communities [63]. Animal feces are the main source of ARGs and pathogenic bacteria in air aerosols. Aerosols carrying microorganisms can travel up to 10 km due to atmospheric dispersion and can also accumulate and settle in dust then be transferred to the skin of workers upon contact [37]. Swine fecal influence on the skin antibiotic resistome is primarily through dust rather than direct contact. This may partly explain the low proportion of swine fecal microorganisms, and collection of air samples in subsequent studies may further determine the contribution of swine fecal microorganisms to microbial changes in the skin. Microbial tracing and strain phylogenetic relationship analysis demonstrates the potential for extensive microbial exchange between workers' skin and their surrounding dust environment. Thus, microbial exchange between the skin and the dust starts when workers enter the swine farm and transient dust microorganisms can remain stable on the workers' skin for 5–10 h until they leave the swine farm at the end of their work (T2).

Previous investigation of the effects of swine farm exposures on the human gut microbiome linked ARG transfers with environmentally mediated microbial transmission events [38]. Similarly skin microbiota shifts in the post‐farm exposure skin correlated with increased abundance and diversity in the antibiotic resistome compared with pre‐farm exposure skin resistome. Previous studies have reported some increase in the relative abundance of ARGs in both the human oral cavity and intestine after occupational exposure in swine farm [38, 64], and our results were similar, particularly in the forehead skin, where the relative abundance of ARGs doubled after 5 h of exposure and remained stable thereafter. This suggests that daily occupational exposure in swine farms can cause enrichment of ARGs in skin, which also suggests that there may be horizontal transfer of ARGs between workers and the swine farm environment with the composition of ARGs in skin after exposure trending towards the dust antibiotic resistome. Our sourcetracker analysis also suggests the environmental dust as the main source of the ARGs, implying ARG acquisition stems from microbiota shifts. The ARG subtypes enriched after occupational exposure belong mainly to the tetracycline, aminoglycoside and MLSB resistance genes. These ARG types have been reported to be commonly found in animal feces and dust from livestock farms [65, 66], and one study analyzing antibiotic resistomes in fresh swine feces samples from three large swine farms in China found a predominance of aminoglycoside, MLSB and tetracycline resistance genes in the pig intestine [67, 68]. Of note, *cfr(A)*, *optrA* and *poxtA* in ARGs significantly enriched after exposure are all phenicol‐oxazolidinone‐tetracycline resistance genes. Their emergence reduces susceptibility to linezolid, flufenicol, and doxycycline which are widely used in veterinary medicines [68], and they have become a major public health challenge. These genes have been reported to be prevalent in *Enterococcus faecalis* isolates from swine farm [69, 70, 71]. The swine farm we studied recorded use of antibiotics includingtylosin gentamicin, amikacin, florfenicol and tetracycline, which relate to the ARG types above. Using these antibiotic as feed additives may have enriched the ARGs and ARB in pig intestines and feces excretion with subsequent dissemination into the air or dust during the movement of animals and handling of feces. This chain of events could explain the enrichment of these ARGs we observed on workers' skin after occupational exposure. One study of airborne bacterial communities and resistomes in a Guangxi province swine farm noted that the resistome mainly contained resistance genes against tetracyclines, aminoglycosides, and lincosamides [72]. The similarity with our results further supports this view. Notably, we identified the *tet*(X) gene with its variants *tet*(X3), *tet*(X4), *tet*(X5) and *tet*(X6) in ARGs enriched after occupational exposure. Tigecycline is a last resort for treating multi‐drug resistant bacterial infections [73], *tet*(X3), *tet*(X4) and *tet*(X5) can significantly reduce the therapeutic efficacy of tigecycline [74, 75, 76, 77]. Although tigecycline is not used in livestock farming, misuse of tetracycline as an animal feed additive has promoted the emergence of *tet*(X) variants. *tet*(X) can be transmitted through bacterial clones and different variants often have different potential bacterial hosts [78]. Studies have shown the emergence of *tet*(X) variants in both human and livestock environments [79, 80]. Spread of *tet*(X) variants could pose a threat to human health via treatment failure in the future, thus monitoring *tet*(X) and its variants in humans, animals, and the environment is critical. Enrichment of *tet*(X) and its variants may be associated with frequent tetracycline use in the studied swine production facility. This implication emphasizes the need for continued improvement of animal husbandry and stewardship practices.

The impact of antibiotic use on human health depends on the connectivity between farm and human associated microbiota. This connectivity includes the potential for ARGs horizontal transfer between animal and human associated bacteria, as well as the transmissibility of ARB in the animal and farm environment to humans [81, 82, 83]. Our analysis indicated high correspondence between post‐farm exposure skin resistomes and the dust resistome, suggesting ARGs were likely acquired from the environment with microbiota shifts. Co‐occurrence network analysis suggests that significantly enriched microorganisms after occupational exposure shared with environment and the skin of workers were potential hosts for significantly enriched ARGs, but additional verification is required [84]. The capture, enrichment, and transmission of ARGs is often due to MGEs, and the transfer of MGEs to a wide range of bacteria, including pathogens and human commensals, is a major cause of persistent transmission of ARGs [85]. We found that 17.40% of contigs carrying ARGs had co‐existing MGEs, and 25 mobile ARGs with different patterns of co‐occurrence in skin and environmental samples were identified among ARGs enriched after occupational exposure, such as *AAC(6’)‐Ie‐APH(2”)‐Ia*, *sul1*, *poxtA*, and *tet*(X), the consistent genetic environment of these mobile ARGs in workers' skin and environmental microbiota provides evidence that important ARGs are shared with human skin and swine farm environmental microbiota. Gene *poxtA* is a mobile ARG we found near insertion sequences *IS*1216E, *IS*S1W, and *IS*Lgar4. *IS*1216E‐mediated translocation is known to contribute to propagation and persistence of the *poxtA* gene in the host [86], and the *IS*1216E‐*poxtA* pattern of mobile ARG was the most abundant.

Metagenomic binning and annotatation of MAGs and ARGs showed a direct correlation. Five species in environmental samples were significantly enriched in skin postexposure. ARGs that demonstrate changes in the skin of swine farm workers after occupational exposure may be carried by microorganisms and transmitted between the skin of workers and the environment after these microorganisms acquire AMR. This study provides a holistic view of the potential ARG hosts, thereby improving the precision of host analysis. Network and metagenomic binning analysis supported that ARG acquisition occurred in concert with microbiota exchange. Furthermore, the findings regarding the stability of microorganisms carrying ARGs on the skin of farm workers postexposure indicates that these organisms could persist on skin. The current mandatory requirement for workers to disinfect before entering the farm, without requiring them to disinfect before leaving the farm, which has the potential to spread ARGs from the farm environment to the surrounding environment and communities. Reassessing the role farm workers and farm management in the One Health framework indicates need for interventions such as requiring workers to wear masks or protective masks while on the job, installing shower disinfection rooms at exits or protocolizing dust vent cleaning to reduce the potential for the spread of ARGs and ARB.

## CONCLUSION

In summary, we show that occupational exposure in a livestock environment dynamically reshaped the skin microbiome and resistome. This remodeling can take place within hours, and the acquired pathogens and ARGs have the potential to expand to the general population using farm workers as an ARG vector. Farmworkers deserve special consideration under the One Health framework to curb spreading antimicrobial resistance.

## METHODS

### Study design and sample collection

We conducted a longitudinal cohort study to investigate the impact of daily occupational exposure on the skin microbiome and antibiotic resistome of farmworkers. The study utilized a swine farm as the experimental group and a school as the control group. The volunteers (age = 45 ± 10 years old, letters: A, B, C, D, E, F, G, H, I, J, K) of the swine farm group (Cohort ID: Workers) were front‐line staff with 1–2 years of experience in a swine farm in Guangxi province, which adopted the closed management mode and had an average of about 20,000 self‐breeding swines. The volunteers (age = 25 ± 3 years old, letters: L, M, N, O, P, Q, R, S, T, U, V) of the school group (Cohort ID: Students) were students from South China Agricultural University. The farm's biosecurity measures require workers to be thoroughly disinfected before entering the farm, to design a study based on actual production process, the control group of students were also asked to have their skin and nasal cavities thoroughly cleaned and sampled at the same time as the farm workers. So the forehead skin swabs and nasal vestibular skin swabs were obtained simultaneously from the two cohorts at four timepoints: T‐1 (8 am: before the workers entering the farm), T0 (cleaning), T1 (5 h after T0), and T2 (10 h after T0). Workers provided dust samples from the production operation area and air vents, as well as swine fecal samples from the delivery and pregnancy room. Students' dust samples were collected from labs and study rooms where the students often work. Each sampling area produced approximately 3–5 samples.

We obtained informed consent from volunteers stating that our reagents were safe before collecting skin samples. We also collected demographic and health history information from volunteers including age, work antibiotic exposures, skin health, and other relevant factors. This information is detailed in Table S1. The Institutional Review Board of South China Agricultural University (SCAU‐IRB) granted approval for the human skin samples in this study.

The swabbing techniques of skin samples we used were described in our previous study [87]. The dust samples are collected by wetting a sterile cotton swab with sterilized saline solution and then wiping it over the sampling surface repeatedly. The collection method of swine fecal sample was to cut off the middle part of the feces with a sterile spoon. All samples were stored at −20°C before DNA extraction.

### DNA extraction

Genomic DNA was extracted from all samples using the QIAamp PowerFecal Pro DNA kit (Qiagen) according to the manufacturer's instructions. The purity and concentration of genomic DNA of environmental samples were first roughly determined using NanoDrop 2000 spectrophotometer (Thermo Fisher Scientific). DNA degradation and contamination were monitored on 1% agarose gels and DNA precise concentration was measured by Qubit® DNA Assay Kit in Qubit® 3.0 Flurometer (LifeTechnologies). The DNA concentration of skin swab samples are generally low, only the precise concentration needs to be measured.

### Metagenomic sequencing and quality control

A library consisting of 350 bp DNA fragments was constructed before sequencing. Each sample was sequenced on the Illumina HiSeq. 4000 platform using a PE150 (paired‐end sequencing) strategy. The raw sequencing reads of each sample were processed independently using Fastp (v0.19.7) [88] for quality control.

### Host DNA removal

Pig and human reference genome database were generated using Bowtie2 (v2.4.5) [89] with the reference genome Sscrofa11.1 (NCBI accession number GCA_000003025.6) and the *Homo sapiens* reference genome GRCh38.p13 (NCBI accession number GCA_000001405.28) respectively. The clean reads obtained after quality control were mapped with reference genome databases using KneadData (v0.10.0) [90] to remove host DNA contamination.

### Quantification of taxa and antibiotic resistome annotation in metagenomic data

MetaPhlAn 3 (v3.0.14) was used to annotate the taxonomy and composition of clean reads. Relative abundance of ARGs was quantified with ShortBRED (v0.9.3) [91] through the integrated Antibiotic Resistance database (CARD, v3.2.4, downloaded July 2022). ShortBRED hits were filtered out if they had counts lower than 2 or mean reads per kilobase of reference sequence per million sample reads (RPKM) lower than 0.001.

### Analysis of common strains and mobile ARGs across hosts

Strain‐level profiling and strain tracking analysis was done using StrainPhlAn3.0 [92]. The resulting alignment was used as input to IQtree (v2.2.0.3) [93] using model selection and 10,000 ultrafast bootstrap replicates to construct phylogenetic trees. All the resulting phylogenetic trees were plotted in iToL [94] online. All the reference genomes were obtained from NCBI website (https://www.ncbi.nlm.nih.gov/). MEGAHIT (v1.2.9) [95] was used to assemble the sequences. ARG annotator (resAnnotator. py) pipeline for annotation of ARGs in contigs obtained is available on GitHub (https://github.com/dantaslab/resAnnotator), and the AGR annotation database we used is the CARD database. Contigs carrying ARGs were extracted and filtered (greater than 500 bp). Use BLASTP to match the ORFs of these contigs against the ISfinder database. Contigs with a distance greater than 5 kb between ARGs and MGEs and overlapping ARGs and MGEs were discarded. The remaining contigs were considered mobile ARGs [38, 96]. The structure for the mobile ARG patterns (the MGE type, ARG type, and length etc) was summarized in Table S2. For mobile ARGs shared by volunteers and the environment, the gene structure was visualized using R package “gggenes”.

### Genome reconstruction and ARG annotation of MAGs

MetaBAT2 (v2.12.1) was used to reconstruct the genome of environmental samples in workers. Bins were dereplicated at 95% average nucleotide identity (ANI) using dRep (v3.4.0) [97], with each MAG being taxonomically equivalent to a microbial species. Completeness and contamination of all bins were assessed using CheckM (v1.2.1) [98] and filtering for completeness ≥50% and contamination ≤10% [99]. The retained MAGs were annotated to the species using the Genome Taxonomy Database Toolkit (GTDB‐Tk) (v2.1.0) [100] and were mapped with the CARD database using BLASTX.

### Statistical analysis

Statistical analysis was performed using the R platform (v4.1.2) [101]. To explore whether the skin microbiome changes significantly after exposure, the “vegan” package [102] was utilized for conducting the NMDS analysis based on Bray‐Curtis distances of various sample groups to observe structural differences of skin microbiome at different time points and the “adonis” function from the “vegan” package was employed to implement PERMANOVA, based on Bray‐Curtis distance and 999 permutations to observe whether the structural differences are significant. Characteristic microbial taxa were analyzed with LEfSe on the Galaxy website (http://huttenhower.sph.harvard.edu/galaxy/, v1.0). The R package “FEAST” was used for bacterial community sourcetracker analysis [103]. To reveal correlation between the ARG profile and microbial community composition, Procrustes analysis was done with the “vegan” package, and significance of the Procrustes statistic was estimated with the protest function using 999 permutations. The visualization of co‐occurrence network diagrams was done using Cytoscape (v3.7.0) [104]. The network visualization of ARG annotation of MAGs and the phylogenetic assignment plot of MAGs were drawn on the interactive platform of Gephi (v0.9.7) [105] and the online website of iToL [94], respectively. The other drawings were all plotted with the R package “ggplot2” [106]. After Benjamini‐Hochberg correction, statistical significance was established at a *p* value (using Student's t‐test) < 0.05.

## AUTHOR CONTRIBUTIONS

Dong‐Rui Chen analyzed the data and wrote the manuscript. Ke Cheng, Lei Wan, Chao‐Yue Cui and Gong Li collected the sample and did the laboratory work. Dong‐Hao Zhao, Yang Yu, Xiao‐Ping Liao and Ya‐Hong Liu contributed to drafting the manuscript. Jian Sun, Xin‐Lei Lian, and Alaric W. D'Souza designed this study. All authors contributed to drafting the manuscript and approved the final draft.

## CONFLICT OF INTEREST STATEMENT

The authors have declared no competing interests.

## ETHICS STATEMENT

The ethics applications (No. 2018G002 and No. 2023‐004) were approved by the Institutional Review Board of South China Agricultural University (SCAU‐IRB) and the Institutional Review Board of Wenzhou Medical University (WMU‐IRB), respectively. All swine feces were sampled under authorization from Animal Research Committees of South China Agricultural University (SCAU‐IACUC). All human skin swab samples were sampled under authorization from the WMU‐IRB.

## Supporting information


**Figure S1.** Changes in microbial diversity (Simpson index) of samples from groups Fh and Ns of two cohorts (A: Farmers and B: Students) at T‐1, T1, and T2. *p* values marked in red mean are less than 0.05.
**Figure S2.** Changes in the relative abundance of significantly varied species.
**Figure S3.** Strain level analysis showing the relationships for human skin and environmental metagenome.
**Figure S4.** Strain level analysis showing the relationships for human skin and environmental metagenome.
**Figure S5.** The alteration of antibiotic resistome structure and diversity across time points.
**Figure S6.** Antibiotic resistome structure in nasal vestibular skin was influenced by occupational exposure.

**Table S1.** Summary information about the questionnaire.
**Table S2.** Shared mobile ARGs and associated MGEs found within human skin and environmental samples.
**Table S3.** Sample collection information.
**Table S4.** Taxonomic profiling of the metagenomic samples.
**Table S5.** Statistical data of the top 5 phyla based on relative abundance of skin samples from each time point.
**Table S6.** Statistical data of the top 10 genera based on relative abundance of skin samples from each time point.
**Table S7.** Statistical data of the top 20 species based on relative abundance of skin samples from each time point.
**Table S8.** Species which up‐/down‐regulated significantly at T1 compared to T‐1 and at T2 compared to T1.
**Table S9.** Characteristics of antibiotics resistance genes in the collected samples.
**Table S10.** ARG subtypes which up‐/down‐regulated significantly at T1 compared to T‐1 and at T2 compared to T1.
**Table S11.** Construction of the swine feces and dust MAGs.

## Data Availability

The metagenomic sequencing data supporting the conclusions of this article have been deposited in the NCBI Sequence Read Archive under the project ID PRJNA982563 (https://www.ncbi.nlm.nih.gov/bioproject/PRJNA982563/). The data and scripts used are saved in GitHub https://github.com/cdr67/Daily-occupational-exposure-in-swine-farm-alters-human-skin-microbiota-and-antibiotic-resistome. Supplementary materials (figures, tables, scripts, graphical abstract, slides, videos, Chinese translated version and update materials) may be found in the online DOI or iMeta Science http://www.imeta.science/.
