# Supplementary material for: Daily occupational exposure in swine farm alters human skin microbiota and antibiotic resistome
Source: Imeta. 2024 Jan 1;3(1):e158. doi: 10.1002/imt2.158 (PMC10989081; doi:10.1002/imt2.158)
Supplement: Supplementary file 2 — Table S1. Summary information about the questionnaire. Table S2. Shared mobile ARGs and associated MGEs found within human skin and environmental samples. Table S3. Sample collection information. Table S4. Taxonomic profiling of the metagenomic samples. Table S5. Statistical data of the top 5 phyla based on relative abundance of skin samples from each time point. Table S6. Statistical data of the top 10 genera based on relative abundance of skin samples from each time point. Table S7. Statistical data of the top 20 species based on relative abundance of skin samples from each time point. Table S8. Species which up‐/down‐regulated significantly at T1 compared to T‐1 and at T2 compared to T1. Table S9. Characteristics of antibiotics resistance genes in the collected samples. Table S10. ARG subtypes which up‐/down‐regulated significantly at T1 compared to T‐1 and at T2 compared to T1. Table S11. Construction of the swine feces and dust MAGs. [file IMT2-3-e158-s001.docx]

**Supporting Information**

**Daily** **occupational exposure in swine farm alters human skin microbiota and** **antibiotic resistome**

**Running title:** Daily exposure in swine farm alters human skin microbiota

Dong-Rui Chen^1,2,3,4,5#^, Ke Cheng^5#^, Lei Wan^1,2,3,4^, Chao-Yue Cui^6^ , Gong Li^1,2,3,4^, Dong-Hao Zhao^1,2,3,4^, Yang Yu^1,2,3,4^, Xiao-Ping Liao^1,2,3,4^, Ya-Hong Liu^1,2,3,4^, Alaric W. D’Souza^7,8*^, Xin-Lei Lian^1,^^2,3*^, Jian Sun^1,2,3,4*^

^1^ State Key Laboratory for Animal Disease Control and Prevention, South China Agricultural University, Guangzhou 510642, PR China

^2^ Guangdong Laboratory for Lingnan Modern Agriculture, National Risk Assessment Laboratory for Antimicrobial Resistance of Animal Original Bacteria, College of Veterinary Medicine, South China Agricultural University, Guangzhou 510642, PR China

^3^ Guangdong Provincial Key Laboratory of Veterinary Pharmaceutics, Development and Safety Evaluation, South China Agricultural University, Guangzhou 510642, PR China

^4^ Jiangsu Co-Innovation Center for the Prevention and Control of Important Animal Infectious Disease and Zoonoses, Yangzhou University, Yangzhou 225009, PR China

^5^ Veterinary Center, Guangxi State Farms Yongxin Animal Husbandry Group Co., Ltd., Nanning 530042, PR China

^6^ Laboratory Animal Centre, Wenzhou Medical University, Wenzhou, China

^7^ Boston Children’s Hospital Department of Pediatrics, Boston, MA, USA

^8^ Harvard Medical School, Boston, MA, USA

**^#^** These authors contributed equally: Dong-Rui Chen, Ke Cheng

***** Correspondence:

[jiansun@scau.edu.cn](mailto:jiansun@scau.edu.cn) (Jian Sun)

[xinlei_lian@scau.edu.cn](mailto:xinlei_lian@scau.edu.cn) (Xin-Lei Lian)

[alaric.dsouza@childrens.harvard.edu](mailto:alaric.dsouza@childrens.harvard.edu) (Alaric W. D’Souza)


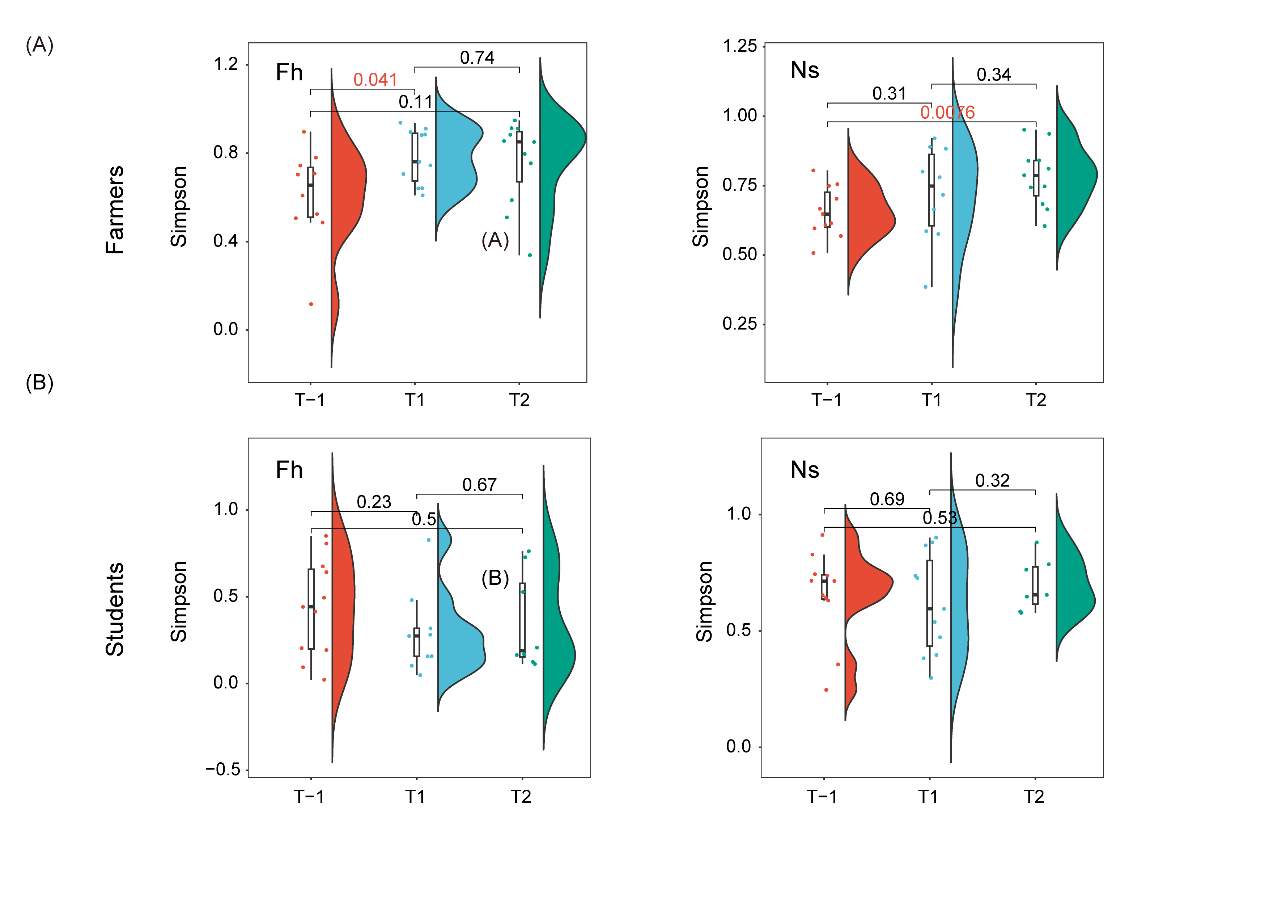


**Figure S1.** Changes in microbial diversity (Simpson index) of samples from groups Fh and Ns of two cohorts (A: Farmers and B: Students) at T-1, T1, and T2. *p*-values marked in red mean are less than 0.05.

**
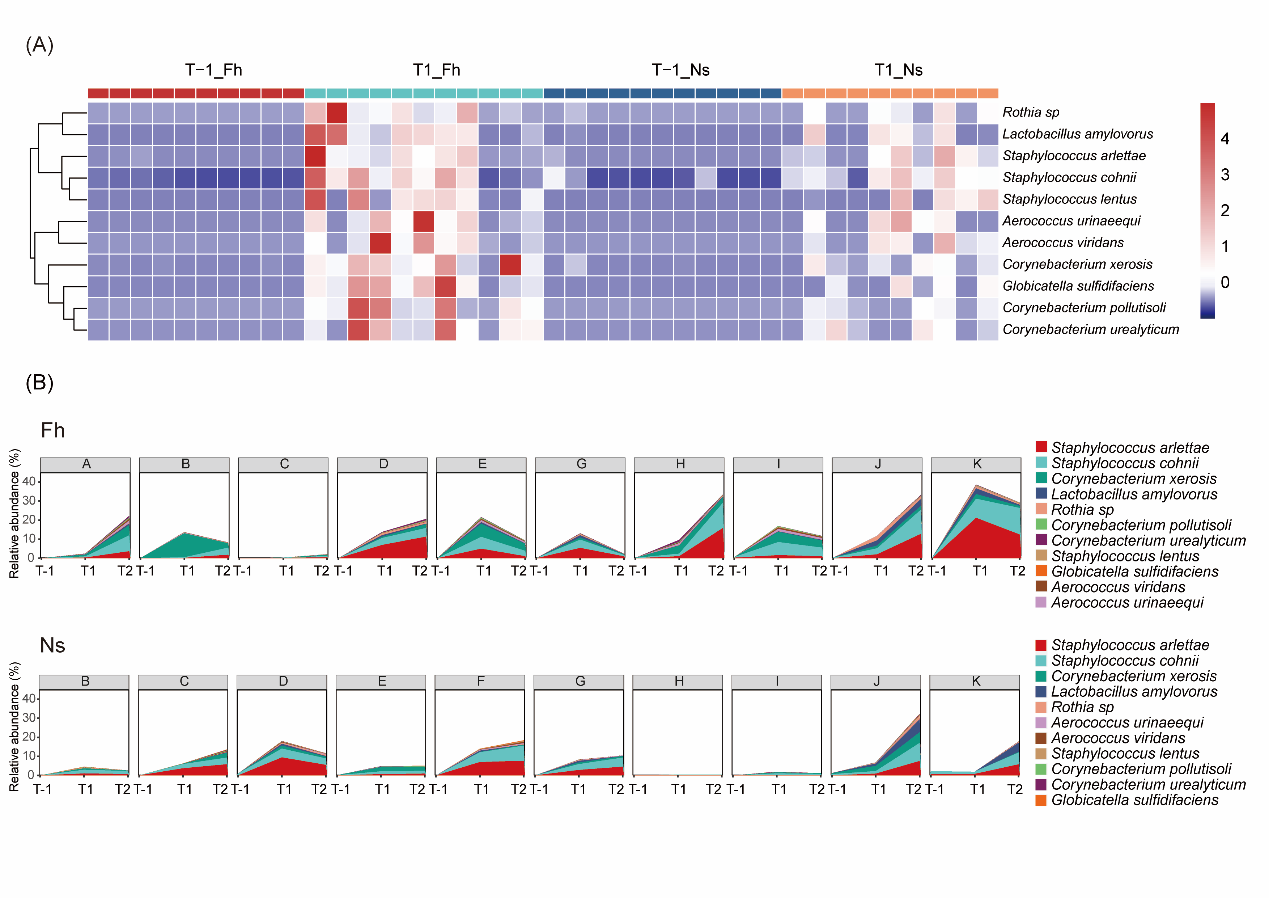
**

**Figure S2.** Changes in the relative abundance of significantly varied species. (A) Heatmap depicting relative abundance of significantly varied species after exposure. Rows (microbial taxa at the species level) are ordered by hierarchical clustering based on Euclidean distance. Values were centered and scaled in the row direction. (B) Changes in the relative abundance of significantly varied species in each individual worker are shown in stacked area plot. A-K are the IDs of the worker volunteers.


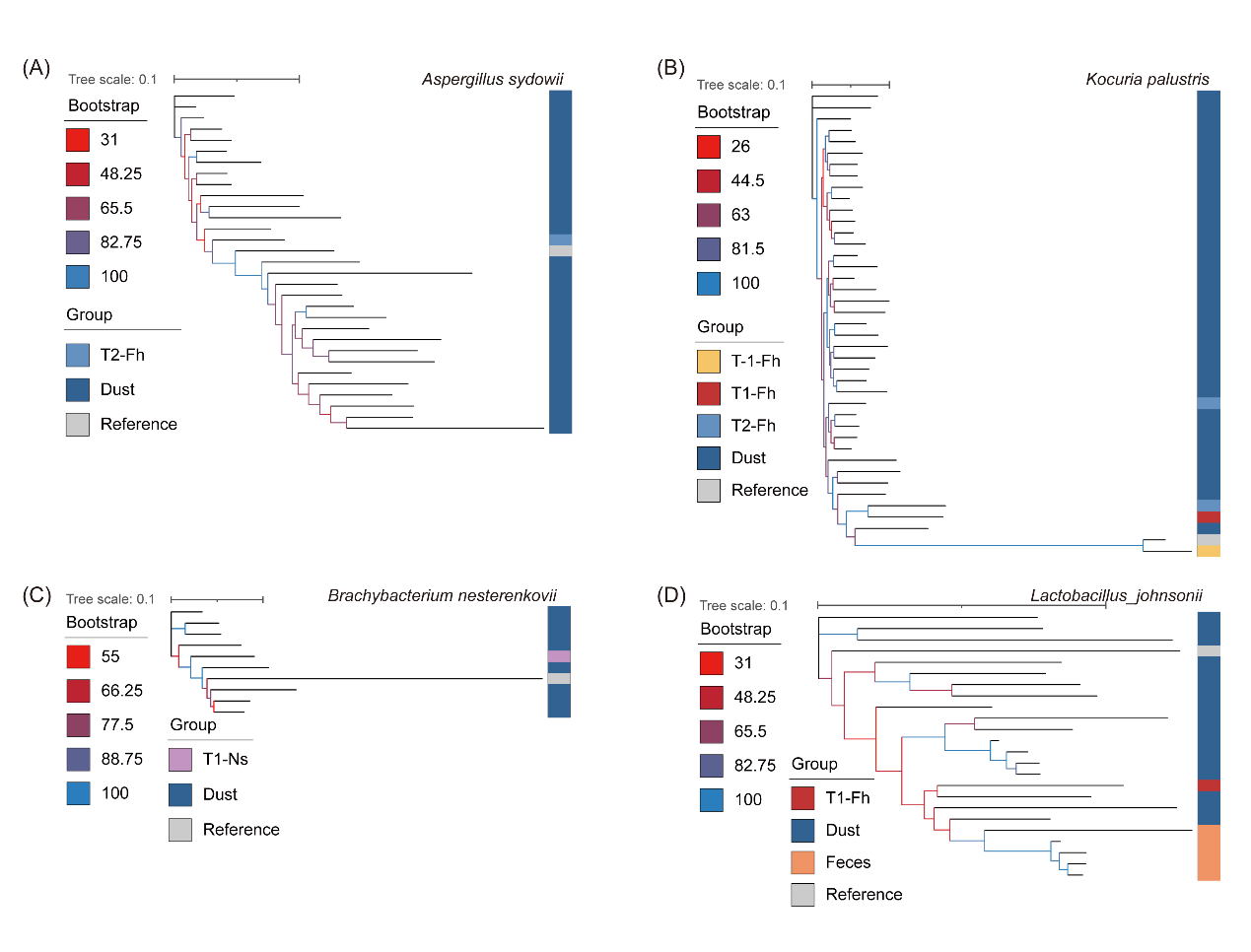


**Figure S3.** Strain level analysis showing the relationships for human skin and environmental metagenome. (A) Phylogenetic tree of *Aspergillus sydowii* strains. The reference genome is *Aspergillus sydowii CBS 593.65*. (B) Phylogenetic tree of *Kocuria palustris* strains. The reference genome is *Kocuria palustris* ASM127534v1. (C) Phylogenetic tree of *Brachybacterium nesterenkovii* strains. The reference genome is *Brachybacterium nesterenkovii CIP104813*. (D) Phylogenetic tree of *Lactobacillus johnsonii* strains. The reference genome is *Lactobacillus johnsonii ASM1484103v1*.


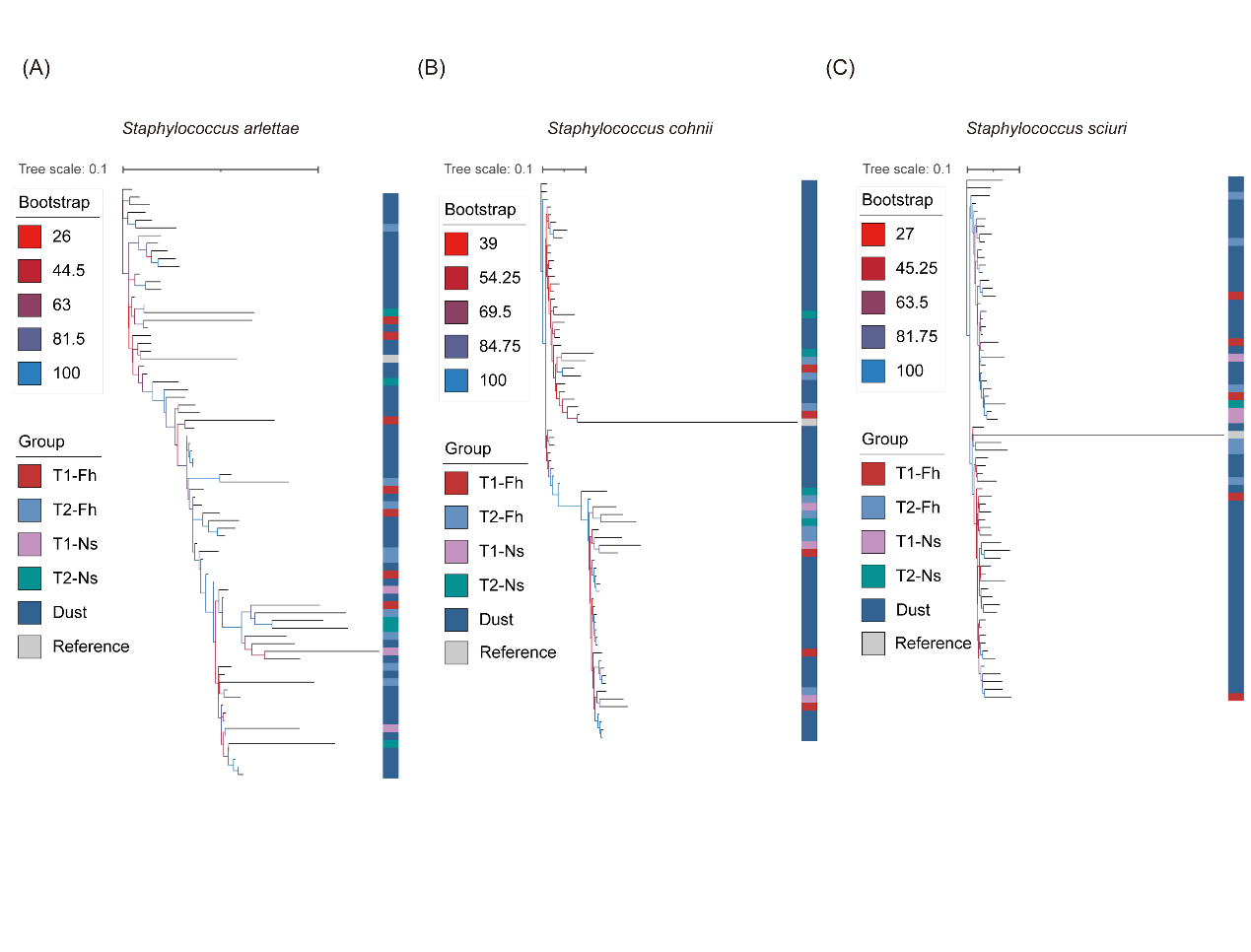


**Figure S4.** Strain level analysis showing the relationships for human skin and environmental metagenome. (A) Phylogenetic tree of *Staphylococcus arlettae* strains. The reference genome is *Staphylococcus arlettae 41556_H01*. (B) Phylogenetic tree of *Staphylococcus cohnii* strains. The reference genome is *Staphylococcus cohnii 44343_D01*. (C) Phylogenetic tree of *Staphylococcus sciuri* strains. The reference genome is *Mammaliicoccus sciuri ASM220916v2.* Bootstrap support values are indicated by the color of legend.

**
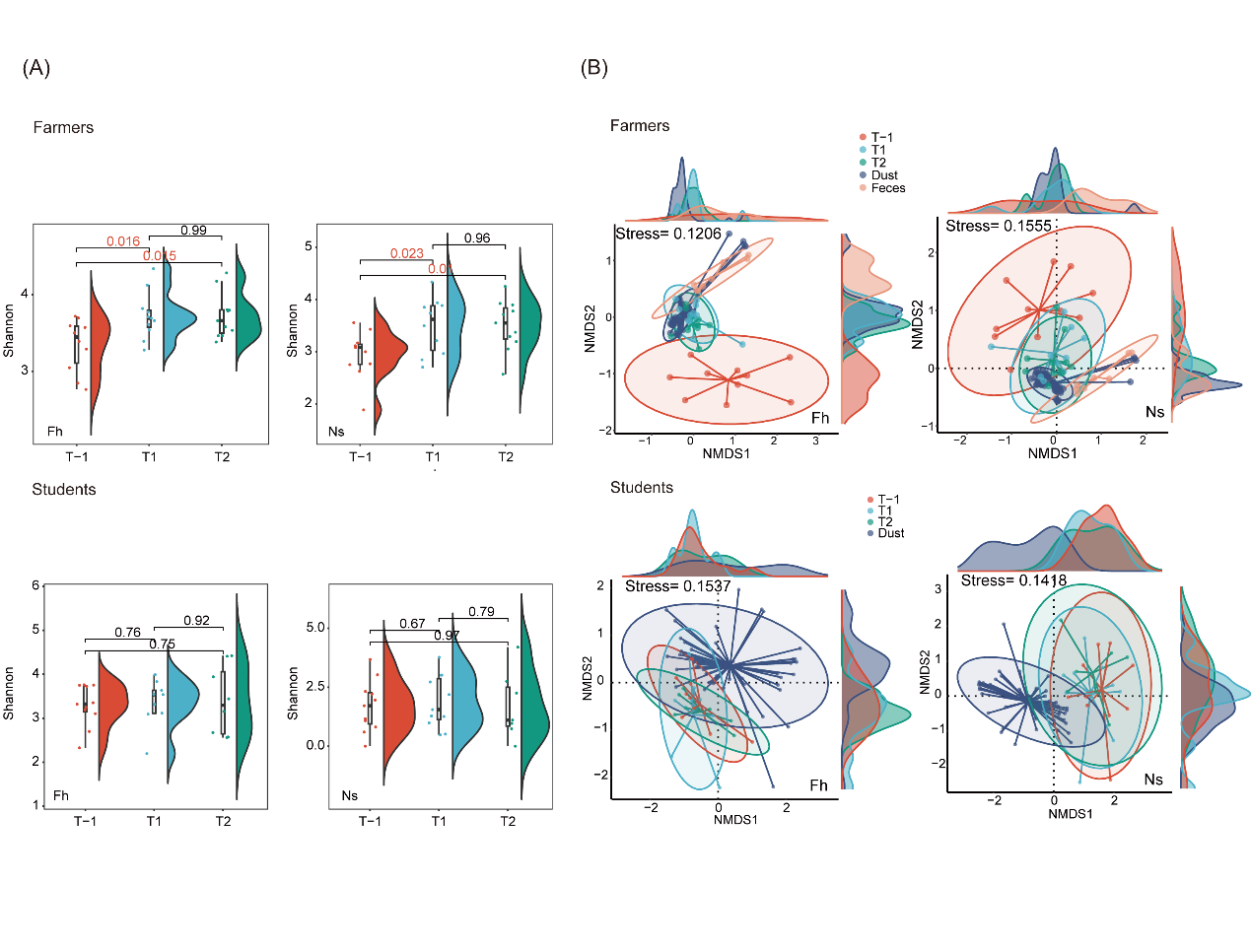
**

**Figure S5.** The alteration of antibiotic resistome structure and diversity across timepoints. (A) Shannon diversity of ARG profiles across three timepoints. Boxes show the distribution of volunteers’ samples (n = 10/11 biologically independent samples per timepoint) (boxes show medians/quartiles; error bars extend to the most extreme values within 1.5 interquartile ranges). *p*-values marked in red are less than 0.05. (B) NMDS analysis of the ARG compositions of human skin and environmental samples in Farmers and Students based on Bray–Curtis dissimilarity.

**
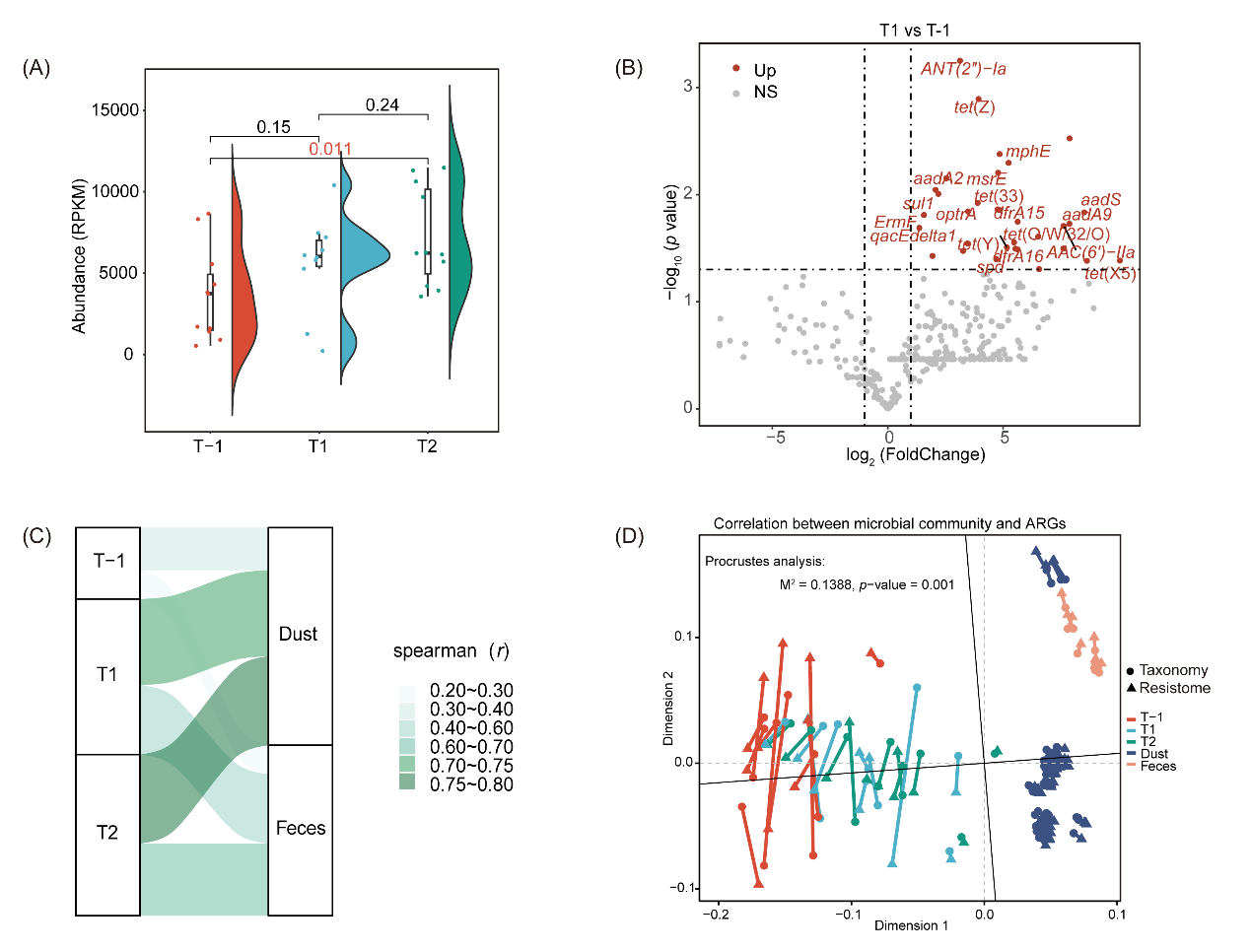
**

**Figure S6.** Antibiotic resistome structure in nasal vestibular skin was influenced by occupational exposure. (A) Sum of abundance of ARGs in nasal vestibular skin across three timepoints. Boxes show the distribution of workers’ samples (n = 10/11 biologically independent samples per timepoint) (boxes show medians/quartiles; error bars extend to the most extreme values within 1.5 interquartile ranges). *p*-values marked in red mean they are less than 0.05. (B) Volcano plots showing the alteration of compositions of ARGs of nasal vestibular skin after 5 hours of occupational exposure. Log_2_FoldChange was used to illustrate the variation of ARGs composition with 5 hours of occupational exposure in swine farm (T1) compared with T-1. The red/blue dots represented the ARGs significantly increased/decreased in T1 compared to T-1. (C) Sankey plot showing the correlation between the ARG composition of nasal vestibular skin across timepoints and the ARG composition of environment. (D) Procrustes analysis connecting the microbiomes and resistomes of microbiota in nasal vestibular skin and environmental samples.
